# The Vitreomacular Interface in Diabetic Retinopathy

**DOI:** 10.1155/2015/392983

**Published:** 2015-09-03

**Authors:** Daniel Agarwal, Rachel Gelman, Claudia Prospero Ponce, William Stevenson, John B. Christoforidis

**Affiliations:** Department of Ophthalmology, University of Arizona Medical Center, Tucson, AZ 85711, USA

## Abstract

Diabetic retinopathy (DR) is a leading health concern and a major cause of blindness. DR can be complicated by scar tissue formation, macular edema, and tractional retinal detachment. Optical coherence tomography has found that patients with DR often have diffuse retinal thickening, cystoid macular edema, posterior hyaloid traction, and tractional retinal detachment. Newer imaging techniques can even detect fine tangential folds and serous macular detachment. The interplay of the vitreous and the retina in the progression of DR involves multiple chemokine and other regulatory factors including VEGF. Understanding the cells infiltrating pathologic membranes at the vitreomacular interface has opened up the possibility of new targets for pharmacotherapy. Vitrectomies for DR remain a vital tool to help relieve tension on the macula by removing membranes, improving edema absorption, and eliminating the scaffold for new membrane formation. Newer treatments such as triamcinolone acetonide and VEGF inhibitors have become essential as a rapid way to control DR at the vitreomacular interface, improve macular edema, and reduce retinal neovascularization. These treatments alone, and in conjunction with PRP, help to prevent worsening of the VMI in patients with DR.

## 1. Introduction

Diabetic retinopathy (DR) is a leading health concern and a major cause of blindness. Worldwide, there are approximately 93 million people with DR, 17 million with proliferative diabetic retinopathy (PDR), 21 million with diabetic macular edema (DME), and 28 million with vision threatening DR [1]. In the United States alone, 4.1 million have DR, with 1 out of 12 suffering from vision threatening DR [2]. DR on exam is characterized by microaneurysms, intraretinal hemorrhages, venous beading, cotton-wool spots, macular edema, neovascularization, retinal ischemia, vitreous hemorrhages, and preretinal scar tissue formation that may lead to tractional retinal detachment [2, 3]. Treatments for macular edema and the complications of neovascularization include focal/grid photocoagulation of retinal tissue, intravitreal therapy with steroid compounds, and agents that block vascular endothelial growth factor (VEGF) as well as surgical intervention for vitreous hemorrhages and repair of tractional formation of retinal detachment.

The role of the vitreomacular interface (VMI) is key in many processes including DR. From macular holes to even influencing age related macular degeneration [4], the VMI plays an outsized role in the emergence and development of several retinal diseases. In DR patients, the VMI can significantly influence the emergence, progression, and response to treatment of DR. Further understanding the vitreomacular interfaces of diabetic retinopathy is warranted in order to better design imaging techniques and treatments to arrest and possibly even reverse progression of DR.

## 2. OCT Imaging of the Vitreomacular Interface

Optical coherence tomography (OCT) has become an increasingly important tool to help better understand the VMI in DR. OCT classification for DME consists of retinal thickness, volume, morphology, diffusion, and epiretinal traction [5]. OCT has found that patients with DME often have diffuse retinal thickening, cystoid macular edema, posterior hyaloid traction, serous retinal detachment, and tractional retinal detachment. Increased retinal thickness, macular edema, and posterior hyaloid traction are associated with worse vision [6]. One study on 9 patients with DME and posterior hyaloid traction found that all patients had retinal thickening, but interestingly 8/9 also had a subclinical shallow macular tractional detachment as well, possibly explaining improved visual acuity after vitrectomy [7].

One study used OCT to examine 48 eyes of patients with persistent DME after at least one session of focal laser treatment. The authors found that 25/48 eyes demonstrated definite VMI abnormalities including vitreoretinal adhesions and epiretinal membrane (ERM). They found that OCT was 1.94 times more sensitive in detecting vitreomacular abnormalities than with standard techniques (slit lamp exam, fluorescein angiography, and fundus photography) [8]. Other studies have found higher detection levels of serous macular detachment with OCT. One study looked at 78 eyes of 58 patients with diabetic cystoid macular edema. Patients were examined with slit lamp exam, fluorescein angiography, and OCT. Serous macular detachment was detected at higher levels than previously known, with OCT allowing for* in vivo* subtle detection of serous macular detachment [9].

Higher resolution OCT imaging, including 3D visualization, has also helped to better visualize the vitreoretinal interface in patients with DR. One study by Abe et al. examined 26 eyes with DME utilizing 3D OCT pre- and postoperatively. The 26 patients were separated into 3 groups: those that had a smooth retinal interface on OCT and 3D imaging, those that had tractional forces only visible on 3D imaging, and those that had an obvious ERM or taut posterior vitreous cortex visible on OCT and 3D imaging. Of the 26 eyes, 11 demonstrated vitreoretinal traction on time domain OCT due to the presence of ERM or a taut posterior hyaloid. 3D imaging of the remaining 15 eyes found that 11 had tangential fine folds [10].

## 3. The Role of Posterior Hyaloid and Vitreous on the Vitreomacular Interface

**Figure 1:**
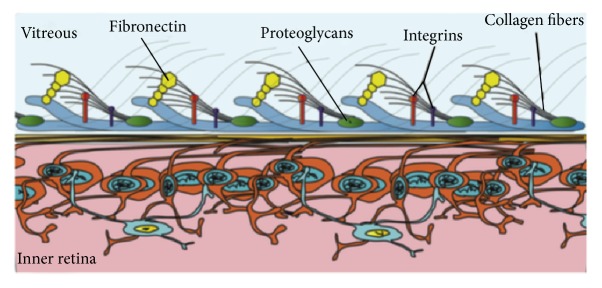
Vitreoretinal attachments at the vitreoretinal interface. Source: [11].

The role of the posterior hyaloid and vitreous in the VMI and the formation of DME has been examined. In normal eyes, the posterior vitreous is attached to the internal limiting membrane (ILM) by collagen at the VMI. Collagen fibers fuse with ILM and help anchor the vitreous cortex to the retina along with laminin, fibronectin, and chondroitin (Figure 1) [11].

Early studies pointed to the vitreous as playing a key role in DME. Nasrallah and colleagues examined the charts of 125 eyes that had undergone a vitreous examination, 105 of which had macular edema. They found a statistically significant relationship between posterior vitreous detachment (PVD) and lack of macular edema, indicating the importance of the vitreous in DME [12]. Another study of 82 diabetic patients with clinically significant macular edema showed that 22 eyes had vitreomacular separation at the onset of the study. Macular edema resolved in 27/82 eyes within 6 months of diagnosis. Interestingly, 12/22 eyes with vitreomacular separation at study onset had spontaneous resolution of their macular edema within 6 months versus 15/60 with vitreomacular adhesion. The authors found that vitreomacular separation led to a statistically significant increase in macular edema resolution [13].

Examining more carefully, one study looked at the vitreoretinal relationship in diabetic patients with and without DME using OCT. Forty-nine eyes of diabetic patients with DME and 49 sex and age matched diabetic control eyes without DME were studied. OCT of the vitreoretinal interface showed that 53% of patients with macular edema had perifoveal PVD, while only 11% of patients without DME had perifoveal PVD. The authors hypothesize that the vitreous may provide traction on the macula during the perifoveal PVD [14]. More recently, swept-source OCT (SS-OCT) was used to examine microstructural tomographic features in proliferative diabetic retinopathy in 4 patients. They were found to have inner and outer layers of vitreoschisis, taut ILM, cortical vitreous separation, and vitreoretinal adhesions [15].

Histologic examination of vitreoretinal tissue was performed in 61 specimens of ILM and epimacular tissue in patients with diffuse DME. Thickened premacular cortical vitreous was found in 47 eyes. Epimacular membrane was found in 23 eyes. Retinal striae and vessel distortion consistent with vitreomacular traction was found in 25 eyes. The authors confirmed a higher incidence of complete PVD in patients with nonproliferative DR versus those with PDR, emphasizing the importance of the vitreous in the development and progression of diabetic retinopathy. Vitreous collagen covered the ILM in 60/61 specimens. PVD in diabetic eyes is likely due to splitting of the vitreous cortex, leaving a layer of collagen on the ILM. Ultrastructure of the VMI in eyes with diffuse DME reveals a layer of vitreous collagen covering the ILM, fibroblasts, and astrocytes embedded in vitreous collagen in prominent premacular cortical vitreous and single or multilayers cell membranes on a layer of vitreous collagen in eyes with vitreomacular traction [16]. Better understanding of the cellular components of the interface will allow for future medical and pharmacologic treatments that may negate the need for vitrectomy in at least some patients.

## 4. Immune and Molecular Pathways Mediating the VMI in DR

Examining the immunocytochemical processes that underlie the VMI in DR is critical in the understanding of the pathophysiology of DR. Electron microscopy of the posterior hyaloid in 2 patients with DME revealed evidence of glial and epithelial cell infiltration. These cells have been implicated in causing tractional forces found in DR. The breakdown in the blood-retina barrier caused by DR may cause increased concentrations of chemoattractants in the vitreous cavity that can then stimulate cell migration [17].

**Figure 2:**
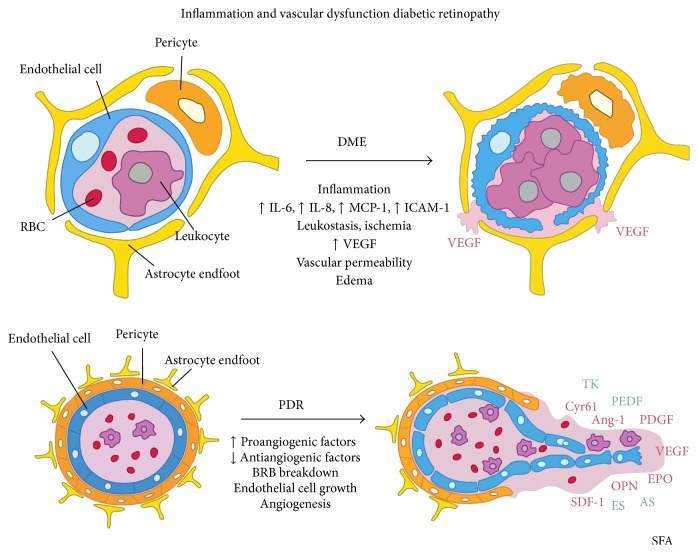
Inflammatory cytokines and their role in PDR. Source: [23].

A study of 30 vitrectomized eyes for DME found VEGF and IL-6 in 8/8 epimacular membranes tested, showing that these molecules may play a role in the development of macular edema [18]. Chemokines including CCL2 have been implicated in inflammation of the diabetic retina, including the activation of retina microglia and macrophages in mice that could lead to disruption of the blood-retina barrier [19]. Examining the relationship between vitreous and PDR more carefully, one study showed the possible role of vitreous levels of IL-8 in deteriorating visual acuity caused by DR, finding that elevated IL-8 levels were independently associated with worse visual outcome [20]. Funatsu and colleagues showed patients with DME had elevated VEGF, ICAM-1, IL-6, and MCP-1 in vitreous fluid, with VEGF and ICAM-1 having a stronger influence on retinal vascular permeability and DME severity [21]. Further studies of vitreous fluid confirmed increased levels of IL-6 and IL-8, as well as elevated levels of IL-1B, VEGF, CCL2, EDN1, and TNF in PDR patients (Figure 2) [22, 23]. Other studies have shown elevated levels of D-serine and glutamate, products believed to be involved in retinal ganglion cell excitotoxicity, in patients with PDR [24].

Kase and colleagues conducted immunohistochemical studies on 16 patients, 13 with PDR and 3 without DM who underwent pars plana vitrectomy (PPV) and ERM peeling. In PDR patients, a statistically significant association was shown between high levels of lymphocyte infiltration into the ERM and poor visual prognosis after vitrectomy because of reproliferation of the ERM [25].

Recently, Dai et al. studied 58 eyes of patients requiring PPV, of which 32 had PDR, to determine the levels of chemokines and growth factors in the vitreous and their relationship with PDR. In non-PDR eyes, levels of 11 chemokines and growth factors tested were similar in patients with macular hole versus those with ERM. However, patients with PDR showed significantly higher levels of 11 chemokines, including CCL17, CCL19, and TGF*β*3. Moderate to strong correlations were also found between VEGF and other mediators. The authors postulate that these chemokines and growth factors could be targeted along with anti-VEGF therapy for PDR treatment [26]. Other chemokines such as IL-18 and serum vascular adhesion protein-1 have been correlated with VEGF levels in patients with DR and could serve as targets for future pharmacotherapy [27, 28].

However, a study of fibrovascular membranes removed from patients with PDR showed more nuanced results. Forty patients with PDR had fibrovascular membranes removed via vitrectomy. T-lymphocytes, B-lymphocytes, and macrophages were found in the fibrovascular membranes, with B-lymphocytes only in active PDR patients. The authors demonstrated a relationship between the density of inflammatory cells and activity of retinopathy. However, they found no association between proinflammatory cells and density of vessels or visual acuity changes postoperatively [29].

## 5. Vitrectomy for DME

Vitrectomy has been shown in some studies to be an effective treatment for DME but chronic changes might still persist [30]. Lewis and colleagues performed PPV with separation of the posterior hyaloid in 10 eyes with DME and thickened, taut posterior hyaloids. Patients had improvement in vision with resolution of macular traction and edema [31]. Ikeda et al. performed vitrectomies on 5 eyes with DME and detached posterior hyaloid membranes, resulting in resolution of DME in 4 of the patients [32]. One study of 30 vitrectomized eyes of patients with DME resulted in statistically significant improvements in visual acuity and reductions in foveal macular edema [18].

Gandorfer and colleagues operated on 12 eyes with diffuse DME, performing PPV with surgical removal of the posterior hyaloid and peeling of the ILM. Retinal thickening improved or resolved in all cases, with visual acuity improvements in 11/12 eyes. Furthermore, no recurrence of macular edema or ERM occurred 8–31 months postoperatively [33]. Their study indicated that not only does vitrectomy release tractional forces on the retina, but also removing the ILM eliminates the scaffold for proliferating astrocytes on the retinal surface. Improved results with peeling of the ILM were also shown by Stefaniotou et al. when they analyzed the surgical outcomes in 73 eyes of 52 patients with DME. In the study, 18 eyes underwent posterior hyaloid membrane removal while 55 eyes underwent ILM peeling as well. More patients that underwent ILM peeling had complete resolution of macular edema than those that just underwent posterior hyaloid removal [34]. Kumagai and colleagues examined ILM peeling in vitreous surgery for DME patients on 135 eyes. Of the 135 eyes, 74 underwent ILM peeling. The authors found that ILM peeling accelerated the absorption of edema in severe DME but did not further improve visual acuity [35].

Another study looked at sixty eyes of patients with chronic DME that underwent pars plana vitrectomy and ILM removal. Reduced leakage within the macula and a decrease in macular thickening were observed in 93% of patients, yet visual acuity improved significantly (2+ lines) in 43% of patients. This suggests that chronic DME may cause structural changes that are difficult to reverse [36]. One study showed that, even without signs of traction on exam, vitrectomy in DME could help to resolve macular edema and improve vision [37]. Another study of 87 eyes with DME and vitreomacular traction found that vitrectomy resulted in reduction in macular edema in most eyes with a more questionable amount of visual acuity improvement. Researchers estimated that 28–49% of patients gained greater than 10 letters' vision improvement while 13–31% demonstrated greater than 10 letters' deterioration [38].

## 6. Retinal Oxygenation

The improvement of macular edema after vitrectomy may be secondary to improved oxygenation of the retina. Primate models have shown that improving systemic oxygenation reduced VEGF mRNA expression in induced ischemic retinas [39]. Vitrectomies performed on rabbit eyes showed a statistically significant increase in oxygen tension of the vitreous that persisted 8 weeks after vitrectomy [40]. Stefansson et al. showed how vitrectomy and lensectomy in cat eyes improve oxygen uptake by the retina from aqueous humor migration [41]. In other states of retinal hypoxia such as induced BRVO in cats, eyes vitrectomized prior to the BRVO event showed no significant change on intraocular oxygen tension, unlike in nonvitrectomized eyes [42].

Studies in humans have also demonstrated an increase in intravitreal oxygen levels after vitrectomy. Holekamp et al. demonstrated that vitrectomy in patients caused a statistically significant increase in oxygen tension both near the lens and in the vitreous. Furthermore, they found that, in patients undergoing repeat vitrectomy, the oxygen tension was significantly higher than in eyes undergoing vitrectomy for the first time, indicating a lasting effect of vitrectomy on ocular oxygen levels [43]. One study analyzing oxygen tension in PDR found that oxygen tension in the midvitreous was 46% lower in PDR patients than in controls, with increased oxygen levels in PDR patients near the posterior pole likely from extensive neovascularization. The study also found upregulation of VEGF in diabetic vitreous, indicating its role in neovascularization [44].

## 7. Retinal Laser Photocoagulation

Retinal laser photocoagulation has been used as a treatment for DME to help reduce visual loss. The Early Treatment Diabetic Retinopathy Study established the efficacy of combination of focal and grid photocoagulation to arrest loss of visual acuity in patients with DR [45]. Yanyali et al. showed that PPV and removal of the ILM were superior to grid laser photocoagulation in the treatment of DME, with greater reductions in foveal thickness and greater improvement in visual acuity [46].

Panretinal photocoagulation (PRP) has been found to be effective especially in combination with other therapies. Yang and colleagues showed that, in high-risk PDR patients, combining intravitreal bevacizumab with PRP provided better short-term regression of retinal neovascularization, rapid clearance of vitreous hemorrhage, and visual improvement. They note that the use of bevacizumab helped to clear the vitreous to allow for more complete laser treatment. In effect, it allows for the rapid onset of bevacizumab to be combined with the more durable effect of laser PRP to provide better visual outcomes and prevent the need for vitrectomy [47]. Tran et al. performed PPV and fibrovascular membrane delamination in 5 patients with PDR, 4 of which had prior PRP. They demonstrated that PRP induces a decrease in ambient mitogen (promitotic signal) and activates apoptosis in diabetic fibrovascular membranes, suggesting an additional mechanism by which PRP helps treat DME [48].

PRP is not without its complications. McDonald and colleagues were one of the earlier groups to report complications from PRP in patients with PDR, noting the most common cause of decreased visual acuity was chronic macular edema and vision loss developing after laser treatment in 8% of eyes. Their study notes that 31 eyes developed posttreatment macular edema but without visual changes [49, 50]. In comparison of weekly versus biweekly PRP treatments for DR, Shimura et al. reported that either frequency did not affect visual acuity but that biweekly treatments allowed for faster recovery of macular thickening after PRP [51].

In addition, Soman and colleagues looked at the effect of PRP on macular morphology in patients with DME but without CSME. They examined 76 eyes of 68 patients and found that 14 eyes had worsened vision 3 months after laser, which the authors believed was secondary to macular edema. All of these patients were reported to have multiple other medical problems such as hypertension, nephropathy, cardiac disease, and dyslipidemia. PRP induced a statistically significant increase in central foveal thickness that persisted for 3 months. Furthermore, 34% of patients with a normal macula suffered morphologic changes after laser including cystoid macular edema, vitreomacular traction, ERM, and subfoveal serous detachment [52]. These patients may require further treatment such as intravitreal injections, further laser, or vitrectomy with membrane removal to control their macular edema.

## 8. Intravitreal Corticosteroids and the Vitreomacular Interface

Intravitreal corticosteroids have been a key tool in the armamentarium against DR and can alter the VMI. Multiple studies have demonstrated the effectiveness of intravitreal triamcinolone acetonide (IVTA) in reducing DME and improving vision in patients with DR [53–58]. Glucocorticoids are believed to inhibit macrophages promoting angiogenesis and ICAM-1 mediating leukocyte adhesion [59–62]. In addition, glucocorticoids help to suppress basement membrane degradation and strengthen tight junctions, both helping to reduce macular edema [59–61, 63]. IVTA has been shown to inhibit the degradation of capillary basement membranes and reduced VEGF and TGF-*β* expression [59, 63, 64].

One study revealed that triamcinolone* in vitro* reduced bovine retinal endothelial cell viability and even induced apoptosis. Triamcinolone* in vivo* caused a reduction in choroidal thickness while downregulating basal expression of COX-2 and VEGF [65]. Increased efficacy of IVTA has been related to elevated baseline levels of IL-8, a proinflammatory cytokine [57]. Uckermann and colleagues discovered that triamcinolone reverses osmotic swelling of Müller glial cells in rat retinas with induced ischemia and inflammation. Triamcinolone stimulates activation of protein kinase A and helps open pathways for K^+^ and Cl^−^ ions to help quickly resolve edema in human patients [66].

**Figure 3:**
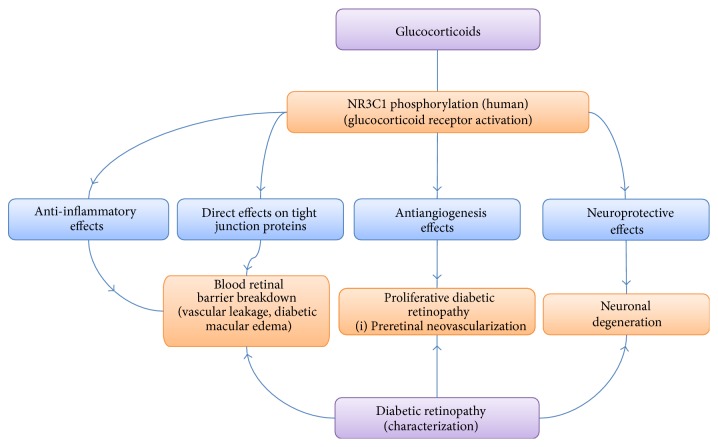
Effects of glucocorticoids on diabetic retinopathy. Glucocorticoids act through several pathways to counteract the negative aspects of DR on the eye. Source: [63].

Lee et al. demonstrated that IVTA reduced central macular thickness in patients with DME. Furthermore, they discovered a correlation between increased intraretinal fluid turbidity and greater reduction in the central macular thickness after IVTA [67]. Interestingly, Sonoda et al. showed that IVTA in DME patients reduced not only central macular thickness, but also subfoveal choroidal thickness lasting 12 weeks [68]. Horii and colleagues examined patients treated with intravitreal or sub-Tenon's injection of triamcinolone for DME with OCT imaging. They found that reflectivity levels of foveal cystoid spaces increased 1 month after triamcinolone administration (*p* = 0.019) but then decreased to baseline levels at 3 and 6 months. The authors show that lower OCT reflectivity in foveal cystoid spaces may signal rebound macular thickening and visual decline in patients treated with triamcinolone for DME [69]. These studies indicate that triamcinolone works on a molecular level to help inhibit inflammation, strengthen tight junctions, and reduce VEGF production in order to improve DME in DR patients (Figure 3).

## 9. VEGF and the Vitreomacular Interface

**Figure 4:**
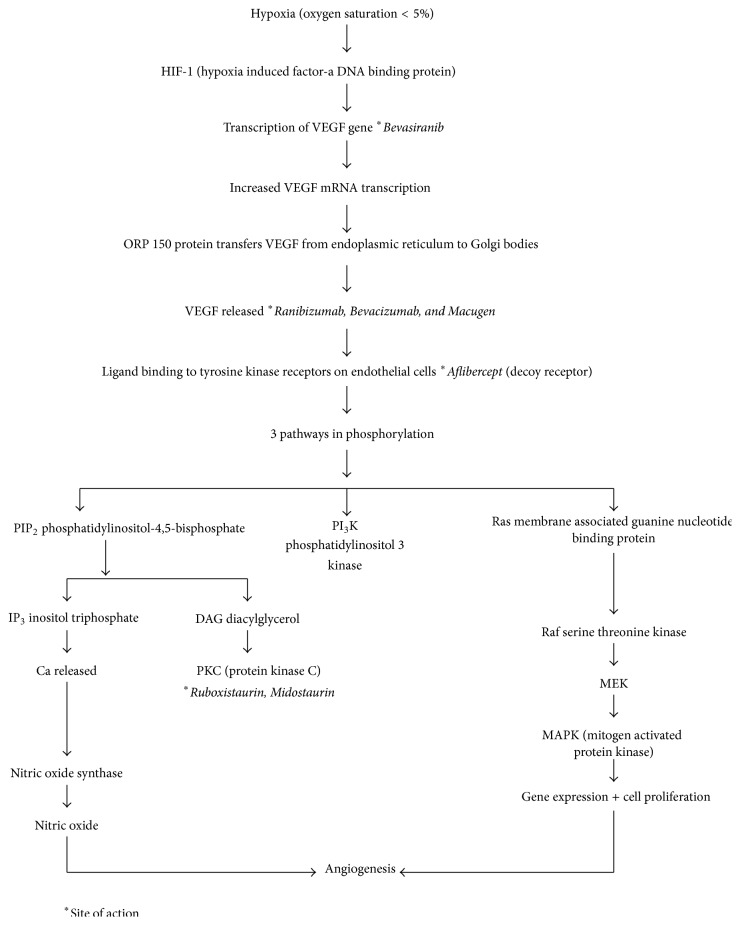
VEGF cascade in retinal hypoxia. Source: [73].

The role of VEGF in influencing the vitreomacular interface has been well investigated. Multiple studies have implicated VEGF in promoting neovascularization of the retina and involvement in PDR (Figure 4) [70–73]. VEGF levels have been shown to decline in response to laser photocoagulation [71]. One study in particular examined preretinal fibrovascular tissue excised during vitrectomy and found that VEGF was expressed in all fourteen patients tested [72]. VEGF levels in vitreous fluid have even been shown to be predictive factors for progression of PDR after vitrectomy in patients with PDR [74]. Chen and colleagues examined ERMs and found that, in PDR patients, 9/11 ERMs stained for VEGF and its receptors. They suggest that an autocrine or paracrine loop may be involved in progression of ERMs [75].

Treatments for retinal neovascularization have included anti-VEGF agents such as bevacizumab. Bevacizumab has been shown to be effective in reducing neovascularization of the retina and resolution of vitreous hemorrhage [76, 77]. Rizzo et al. reported the efficacy of preoperative treatment (5–7 days before surgery) with bevacizumab in patients undergoing pars plana vitrectomy for complications of PDR. They demonstrated that surgical time and intraoperative bleeding were both reduced in patients with preoperative PPV, indicating the rapid regression of neovascularization in the retina [78]. On a molecular level, bevacizumab was shown by Suzuki et al. to reduce not only VEGF, but also other inflammatory cytokines including IL-1RA, IL-5, IL-10, IL-12, IL-13, and interferon-*γ* [79]. A 2014 study found that, in patients injected multiple times with anti-VEGF treatments, patients with vitreomacular interface abnormalities such as ERMs or vitreomacular adhesions had less change in best-corrected vision than those with only DME after 3 injections. This indicates a possible role of vitreomacular interface abnormalities in reducing the therapeutic effects of anti-VEGF agents [80].

## 10. Conclusion

In conclusion, there are multiple factors at work in the vitreomacular interface including ERM, taut posterior cortices, vitreoschisis, PVD, and adhesions. Evidence of glial cells, collagen, fibroblasts, astrocytes, and retinal pigment epithelial cells has been found on either the hyaloid, cortical vitreous, or the ILM. Interestingly, complete PVDs seem to improve macular edema in some cases, possibly by reducing traction. Factors such as CCL2, IL-6, IL-8, IL-18, and VEGF may also play roles in altering the vitreomacular interface by increasing edema, encouraging neovascularization, and worsening visual outcome. Multiple treatments that alter the VMI, including ILM/posterior hyaloid peeling, PRP, triamcinolone acetonide, and VEGF inhibitors, have been shown to help in various degrees to arrest the progression of PDR and/or improve vision. Overall, there are multiple elements and significant interplay in the vitreomacular interface of diabetic retinopathy.
